# The Impact of Osteopontin and Galectin-7 on the Preoperative Diagnosis of Ovarian Tumors: A Case–Control Study

**DOI:** 10.3390/jcm15062178

**Published:** 2026-03-12

**Authors:** Foteini Chouliara, Aikaterini Sidera, Ioannis Tsakiridis, Areti Kourti, Georgios Michos, Evangelos Papanikolaou, Themistoklis Dagklis, Apostolos Mamopoulos, Kali Makedou, Ioannis Kalogiannidis

**Affiliations:** 1 Third Department of Obstetrics and Gynecology, School of Medicine, Faculty of Health Sciences, Aristotle University of Thessaloniki, 541-24 Thessaloniki, Greece; foteinichouliara3@gmail.com (F.C.); katerinasid96@hotmail.com (A.S.); gmichos@auth.gr (G.M.); evpapanikolaou@auth.gr (E.P.); dagklis@auth.gr (T.D.); amamop@auth.gr (A.M.); ikalogia@auth.gr (I.K.); 2 Laboratory of Biological Chemistry, School of Medicine, Aristotle University of Thessaloniki, 541-24 Thessaloniki, Greece; aretikourti@auth.gr; 3 Laboratory of Biochemistry, AHEPA University Hospital, School of Medicine, Aristotle University of Thessaloniki, 541-24 Thessaloniki, Greece; kmakedou@auth.gr

**Keywords:** ovarian cancer, ovarian tumors, pre-operative diagnosis, tumor biomarkers, osteopontin, galectin-7

## Abstract

**Background/Objectives**: Accurate preoperative discrimination between women with ovarian pathology and healthy controls, as well as between benign and malignant ovarian tumors, remains challenging. This study aimed to evaluate the usefulness of osteopontin and galectin-7 on the diagnosis of ovarian tumors. **Methods**: This prospective single-center case–control study was conducted at the Third Department of Obstetrics & Gynecology, School of Medicine, Faculty of Health Sciences, Aristotle University of Thessaloniki, Greece, between 2018 and 2024. Preoperative serum levels of osteopontin, galectin-7, and established tumor markers (CA-125, CA19-9, CA15-3, CEA, AFP) were analyzed. Biomarker distributions were compared using non-parametric tests. Associations with clinical variables were explored using correlation analyses. Logistic regression and receiver operating characteristic (ROC) curve analyses were performed to assess diagnostic performance. **Results**: The study population included 116 women: 52 healthy controls, 45 patients with benign ovarian tumors, and 19 patients with malignant ovarian tumors. Serum osteopontin and galectin-7 levels did not differ significantly between control and study group (*p* = 0.562 and *p* = 0.138, respectively), nor between benign and malignant tumors (*p* = 0.784 and *p* = 0.140, respectively). Osteopontin showed no discriminatory ability (AUC = 0.47), while galectin-7 demonstrated weak discrimination (AUC = 0.63). A combined model yielded modest improvement (AUC = 0.69), remaining below clinically meaningful thresholds. CA-125 was the only biomarker significantly associated with malignancy (OR = 1.03, *p* = 0.038). Galectin-7 levels were higher in premenopausal women and inversely correlated with age, suggesting demographic rather than malignant influence. **Conclusions**: Despite strong biological relevance, circulating osteopontin and galectin-7 did not provide meaningful diagnostic discrimination between women with ovarian pathology and healthy controls or between benign and malignant ovarian tumors. CA-125 remained the most informative serum marker in this setting. Future efforts should focus on multi-marker strategies integrated with imaging and clinical assessment.

## 1. Introduction

Ovarian cancer remains one of the most lethal gynecological malignancies worldwide, often referred to as a “silent killer” [1]. In 2020, there were approximately 314,000 new cases and 207,000 deaths attributed to ovarian cancer globally, making it the eighth most common cause of cancer-related mortality among women [2]. The majority of malignant ovarian tumors are epithelial in origin, accounting for more than 90% of the diagnosed cases [3]. Due to their indolent presentation, absence of effective early screening, and a unique metastatic process, most ovarian cancer cases are diagnosed at late stages (FIGO stage III or IV), where the five-year survival rate is around 30% [4,5]. By contrast, early stage detection (FIGO stage I-II) dramatically improves outcome, with survival exceeding 70%, thus underscoring the need for improved preoperative risk stratification [4].

Preoperative evaluation of adnexal masses currently relies on a combination of clinical examination, imaging, and serum biomarkers; CA-125 is the most widely used tumor marker, but it lacks specificity and may also be increased in numerous benign conditions, including endometriosis, fibroids, pelvic inflammatory disease, and even normal menstruation [6]. Multivariable algorithms, i.e., Risk of Malignancy Index (RMI), have been developed to improve diagnostic performance, although their accuracy varies with menopausal status, age, and tumor histology [7,8,9]. Additional tumor markers such as CA19-9, CA15-3, carcinoembryonic antigen (CEA), and alpha-fetoprotein (AFP) may aid in selected tumor subtypes, particularly mucinous or germ-cell tumors, but have limited value as general diagnostic tools [10,11,12,13,14].

Osteopontin and galectin-7 have emerged as potential candidate biomarkers due to their roles in biological processes central to ovarian tumorigenesis, including tumor-stromal interaction, immunoregulation, invasion, and metastatic progression [15,16,17,18]. Elevated circulating osteopontin levels have been reported in ovarian cancer compared with benign disease and healthy controls, and several studies have explored its diagnostic characteristics [19,20,21,22]. Moreover, Galectin-7, a β-galactoside-binding lectin predominantly expressed in epithelial tissues, has been associated with tumor progression and survival in ovarian cancer, although evidence is largely derived from tissue-based studies and data on circulating levels remain limited [23,24].

Given the diagnostic challenges posed by adnexal masses and the need for improved biochemical markers, this study investigates the serum levels of osteopontin and galectin-7 in women with benign and malignant ovarian tumors, alongside a healthy control group. The primary objective was to determine whether ovarian pathology is associated with altered concentrations of these biomarkers. As a secondary objective, we evaluated whether osteopontin and galectin-7 demonstrate a discriminatory potential between benign and malignant tumors and assessed their diagnostic performance in comparison with established tumor markers commonly used in clinical practice.

## 2. Material & Methods

### 2.1. Study Design and Population

This prospective, single-center case–control study was conducted at the Third Department of Obstetrics & Gynecology, School of Medicine, Faculty of Health Sciences, Aristotle University of Thessaloniki, Greece, between 2018 and 2024.

The study group included women diagnosed with an ovarian mass undergoing abdominal surgery. Clinical and demographic variables included age, body mass index (BMI), menopausal status, smoking history, hypertension, diabetes mellitus, thyroid disorders, and history of concurrent or prior malignancy were documented. Tumor marker levels measured pre-operatively included CA-125, CA 19-9, CA 15-3, carcinoembryonic antigen (CEA), alpha-fetoprotein (AFP), galectin-7, and osteopontin. Histopathology reports were used to classify tumors as benign or malignant according to the final postoperative diagnosis. Benign lesions were categorized by histological subtype, and malignant tumors were classified according to histological subtype and FIGO stage. Details regarding surgical procedure, recurrence status, and survival outcomes were also recorded.

The control group consisted of asymptomatic women without known ovarian or adnexal pathology. Eligibility required an ultrasound-confirmed absence of adnexal masses, as documented by a transvaginal pelvic ultrasound performed before enrollment. Controls were recruited across a broad age range and had no symptoms suggestive of gynecologic disease. Each participant underwent venipuncture for the collection of a serum sample, which was analyzed for osteopontin and galectin-7 using the same laboratory procedures applied to the patient cohort. This ensured methodological consistency and allowed direct comparison between healthy controls and women with adnexal pathology.

### 2.2. Biomarker Measurement

Serum samples were obtained preoperatively, processed according to institutional laboratory protocols, and analyzed using validated immunoassay techniques. Osteopontin and galectin-7 concentrations were quantified from serum using commercially available ELISA kits by Boster Biological Technology (Pleasanton, CA, USA). CA-125, CA19-9, CA15-3, CEA, and AFP were analyzed using Electrochemiluminence immunoassay (ECLIA) run by COBAS 8000, Roche Diagnostics modular analyzer series (Manheim, Germany). All biomarker values were entered into a unified dataset and checked for completeness and extreme outliers.

### 2.3. Statistical Analysis

Descriptive statistics were used to present demographic and clinical characteristics. Continuous variables were summarized as mean ± standard deviation (SD) or median with range, as appropriate were compared between groups using Student’s *t*-test. Categorical variables were summarized as counts and percentages and were compared using Pearson’s chi-squared test or Fisher’s exact test, as appropriate when expected cell counts were small. To evaluate differences in osteopontin and galectin values across demographic and other characteristics (2 categories’ comparison), the Mann–Whitney U test was used. To assess whether biomarker levels differed across three clinical categories (Healthy controls, Benign tumors, Malignant tumors), Kruskal–Wallis tests were conducted. Spearman’s rank correlation coefficients were calculated to explore associations between osteopontin, galectin-7, and clinical or biochemical variables including CA-125, CA19-9, CA15-3, CEA, AFP, age, and BMI. To investigate a relationship between each biomarker and malignancy, logistic regression was used. The predictor variables osteopontin, galectin-7, CA-125, CA-19-9, CA15- 3, CEA, and AFP were the coefficients in the model. Odds ratios (ORs) with 95% confidence intervals (CIs) were reported to quantify the strength of association. The diagnostic performance of individual biomarkers and the combined logistic model was evaluated using receiver operating characteristic (ROC) curve analysis. Optimal cut-off points were explored using Youden’s index, although these were interpreted cautiously due to low discriminatory performance. For the comparative analysis of serum biomarkers (CA-125, CA19-9, CA15-3, CEA, and AFP), participants were grouped into two categories: non-malignant (including healthy controls and benign ovarian tumors) and malignant ovarian tumors. Biomarker levels were compared between these two groups using the Mann–Whitney U test. Analyses according to individual benign histological subtypes were not performed due to the limited number of cases within each category. Distributions of tumor markers were visualized using boxplots (and jittered points for individual values). For the analysis R (version 4.4.1) and Rstudio were used. The significance level was set at *p* < 0.05.

### 2.4. Ethics

Ethical approval was obtained from the Bioethics Committee of the Aristotle University of Thessaloniki, Greece (No 1/21 November 2018). Informed consent was obtained from all participants. No incentives were provided for participation in this study.

## 3. Results

A total of 116 women were included in the study, comprising 52 healthy controls, 45 cases with benign ovarian pathology, and 19 cases with malignant ovarian tumors. Age and BMI were comparable between controls and cases (mean age 44.3 vs. 47.8 years, *p* = 0.167; mean BMI 26.6 vs. 25.9 kg/m^2^, *p* = 0.562), while certain health characteristics differed: cases were more frequently postmenopausal and hypertensive and had a higher prevalence of prior childbirth compared with controls (*p* < 0.05 for all) (Table 1 and Table 2).

Demographic and clinical characteristics of the patient group are summarized in Table 3. Among cases, benign cases mainly included serous and mucinous cystadenomas, mature cystic teratomas, endometriomas, and fibromas. Malignant cases consisted of serous cystadenocarcinoma, mucinous carcinoma, clear-cell carcinoma, endometrioid carcinoma, borderline tumors, and Brenner tumors (Appendix A, Table A1 and Table A2).

The distribution of FIGO stages among malignant ovarian tumors is presented in Table 4. The majority of cases were early-stage disease (63.0% FIGO stage I), while more advanced-stage tumors (FIGO III-IV) accounted for 26.3% of cases. Most cases had no prior cancer and no family history of malignancy. Surgical management is presented in Appendix A, Table A3.

### 3.1. Tumor Biomarkers

Serum osteopontin and galectin-7 levels were compared between controls and cases, and no statistically significant differences were observed between the two groups for either biomarker. Osteopontin levels were comparable between controls and cases (*p* = 0.562), as were galectin-7 levels (*p* = 0.138), despite considerable variability in both markers (Table 5).

**Figure 1:**
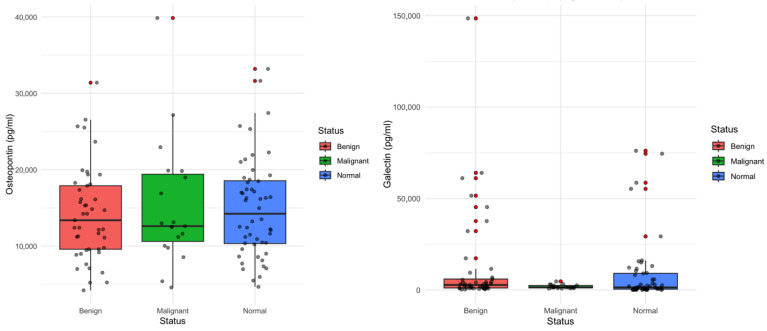
Distributions of osteopontin and galectin-7 in controls (normal) vs. cases (benign and malignant). No statistically significant differences were observed between groups for osteopontin (*p* = 0.562) or galectin-7 (*p* = 0.138).

Furthermore, serum osteopontin concentrations did not differ significantly among healthy controls, cases with benign ovarian tumors, and cases with malignant ovarian tumors (Table 6). Median osteopontin levels were similar across groups (14,226 pg/mL in controls, 13,376 pg/mL in benign tumors, and 12,604 pg/mL in malignant tumors), with substantial overlap in distributions (Kruskal–Wallis *p* = 0.784; Figure 1). Galectin-7 exhibited greater variability than osteopontin, with several high outliers observed in the benign group. Median galectin-7 levels were also similar among healthy, benign, and malignant participants (1376 pg/mL, 2719 pg/mL, and 1421 pg/mL, respectively). Although benign tumors tended to exhibit higher galectin-7 values, these differences did not reach statistical significance (Kruskal–Wallis *p* = 0.140; Figure 1).

### 3.2. Associations with Clinical and Demographic Variables

**Figure A1:**
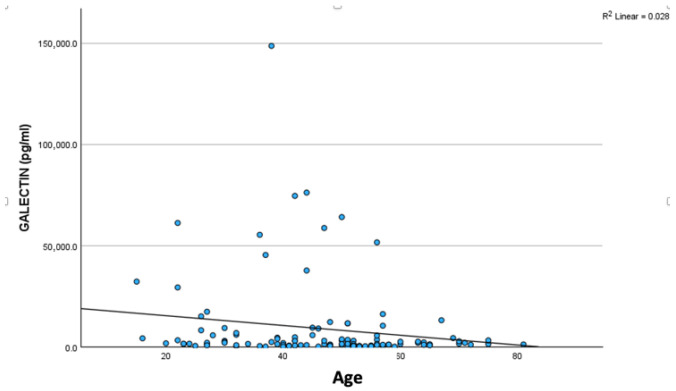
Galectin-7 levels in relation to age.

Osteopontin levels were not significantly associated with age, menopausal status, BMI, smoking, hypertension or diabetes. In contrast, galectin-7 levels demonstrated a significant association with menopausal status and age. Premenopausal women had higher galectin-7 concentrations than postmenopausal women (*p* = 0.023), and galectin-7 levels were negatively correlated with age (Spearman’s rho = −0.220, *p* = 0.018; Appendix A, Figure A1). No significant associations were observed between galectin-7 and BMI, smoking, hypertension, diabetes, parity, or cancer history (*p* > 0.05) (Appendix A, Table A4).

### 3.3. Correlation with Established Tumor Markers

Correlation analysis further confirmed that osteopontin and galectin-7 are largely independent of conventional tumor markers such as CA-125, CA19-9, CA15-3, CEA, and AFP (all *p* > 0.05) (Table 7).

### 3.4. Logistic Regression and Diagnostic Performance

**Figure 2:**
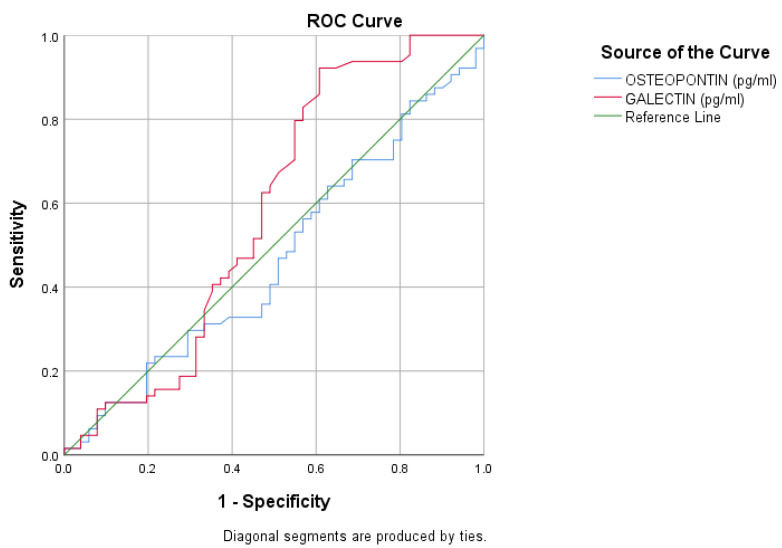
Receiver operating characteristic (ROC) curves for osteopontin and galectin-7.

Inspection of the ROC curve indicated that neither biomarker was able to effectively discriminate between cases and controls. This finding was confirmed by the statistical estimation of the area under the curve (AUC), which was 0.468 (95% CI: 0.362–0.575; *p* = 0.562) for osteopontin and 0.581 (95% CI: 0.469–0.693; *p* = 0.138) for galectin-7, indicating no statistically significant discriminatory performance for either marker (Figure 2; Appendix A, Table A5).

**Figure 3:**
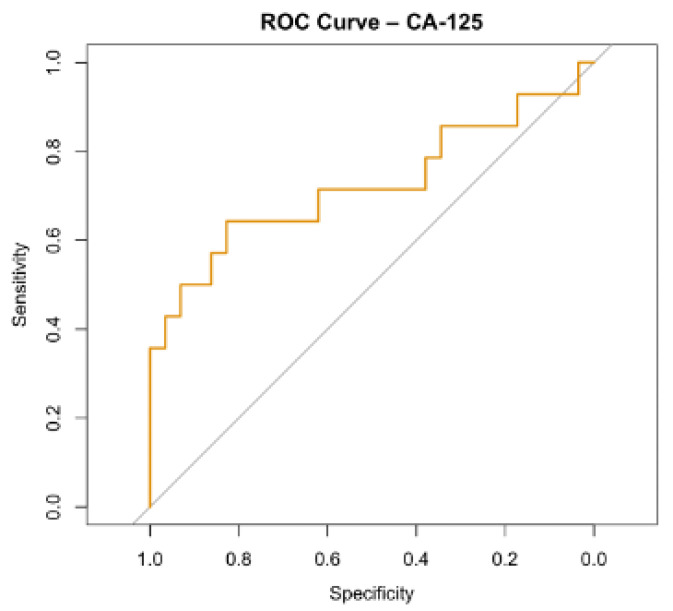
ROC curve for CA-125.

Logistic regression analysis evaluating the association between individual biomarkers and malignancy demonstrated that CA-125 was the only marker significantly associated with malignant ovarian tumors (odds ratio [OR] = 1.03, *p* = 0.038). The ROC curve for CA-125 is presented in Figure 3.

**Figure 4:**
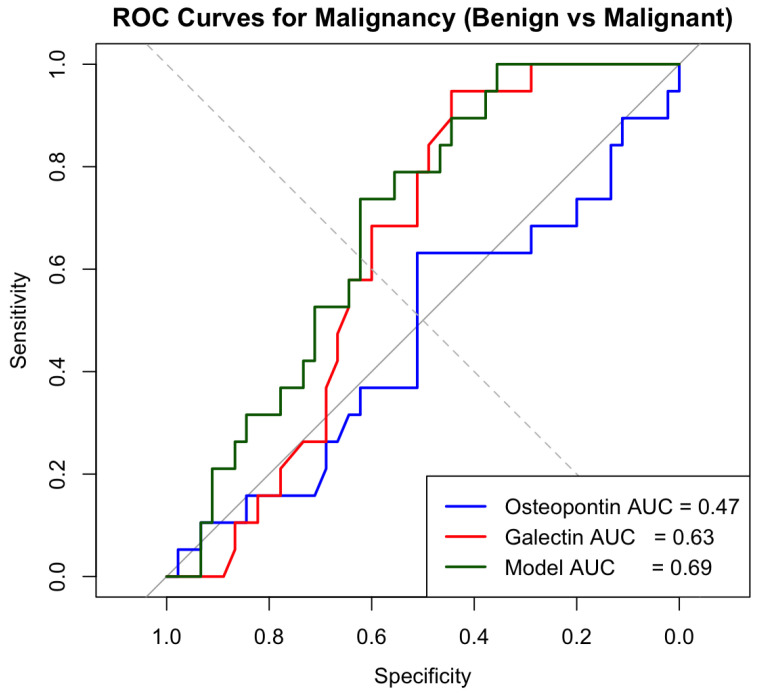
Receiver operating characteristic (ROC) curves for osteopontin (blue), galectin-7 (red), and the combined logistic regression model (green) in discriminating malignant from benign ovarian tumors.

Logistic regression analysis demonstrated that neither osteopontin nor galectin-7 significantly predicted malignant pathology among cases with ovarian masses. In a model comparing malignant to benign tumors, osteopontin showed no meaningful association with malignancy (OR = 1.00005, 95% CI: 0.99996–1.00014, *p* = 0.259). Galectin-7 exhibited a non-significant inverse trend (OR = 0.99971, 95% CI: 0.99924–0.99995, *p* = 0.132), reflecting its higher concentrations in benign lesions. Both biomarkers had odds ratios extremely close to 1.0, indicating negligible effect sizes, and their confidence intervals overlapped unity. ROC curve analysis confirmed the limited diagnostic value of the investigated biomarkers. Osteopontin demonstrated no discriminatory ability (AUC = 0.47), while galectin-7 showed weak discriminatory ability (AUC = 0.63), while a combined logistic model incorporating both biomarkers yielded only small improvement (AUC = 0.69) (Figure 4).

### 3.5. Ca-125, Ca 19-9, Ca 15-3, CEA, and AFP

**Figure 5:**
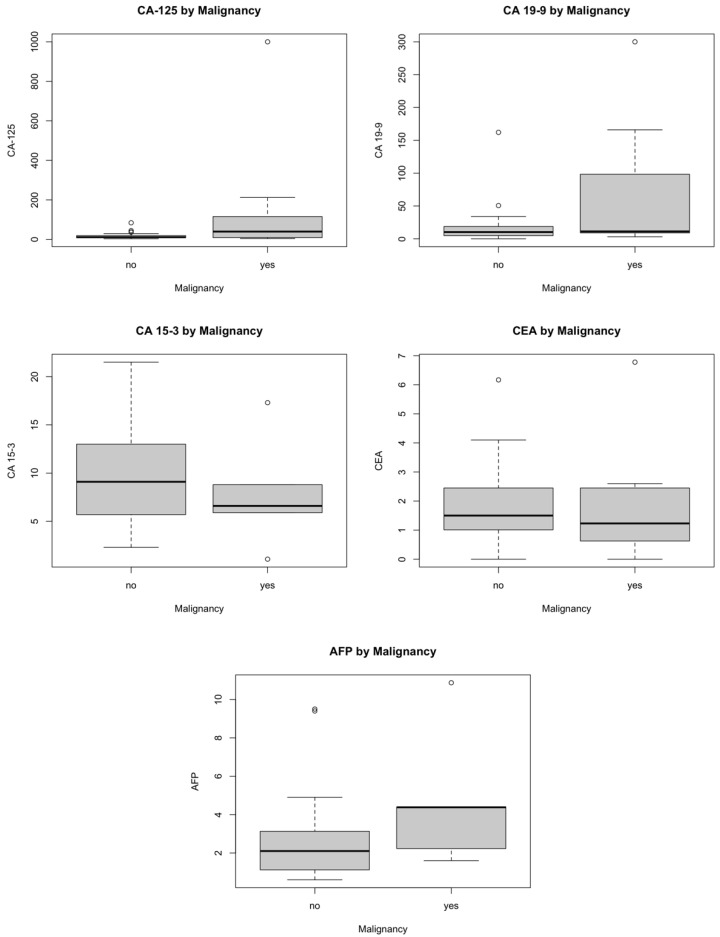
Distribution of biomarkers among benign and malignant cases. A statistically significant difference was observed only for CA-125 (*p* = 0.019), while no significant differences were found for CA19-9 (*p* = 0.326), CA15-3 (*p* = 0.490), CEA (*p* = 0.648), or AFP (*p* = 0.190).

Women with malignant tumors had significantly higher CA-125 values (median 39.5 vs. 10.9 U/mL, Mann–Whitney U test, *p* = 0.019). Preoperative tumor markers varied widely among cases. CA-125 showed a broad range (2.8–1000 U/mL), with a mean of 53.4 ± 154.7 U/mL, while CA19-9 (Mann–Whitney U test, *p* = 0.326) and CA15-3 (Mann–Whitney U test, *p* = 0.490) demonstrated lower variability. AFP (Mann–Whitney U test, *p* = 0.190 and CEA (Mann–Whitney U test, *p* = 0.648) values remained low and were within expected clinical ranges for non-hepatic or non-gastrointestinal malignancies. The variance and distribution of all markers were demonstrated visually using standardized jitter plots (Figure 5).

## 4. Discussion

In this study, among all biomarkers assessed, CA-125 was the only marker independently associated with malignancy. In contrast, osteopontin and galectin-7 did not differ significantly between diagnostic groups and demonstrated limited discriminatory ability in both logistic regression and receiver operating characteristic analyses. These results demonstrate that, despite strong biological plausibility, neither osteopontin nor galectin-7 provided clinically meaningful diagnostic values in the preoperative evaluation of adnexal masses in this study population.

Several factors may explain these findings. First, the malignant group of patients was relatively small (n = 19) and predominantly consisted of early-stage tumors (63.0% FIGO stage I). Given that circulating biomarker levels often correlate with tumor burden, the limited representation of advanced-stage disease may have reduced the likelihood of detecting significant systemic elevations. Second, the malignant cohort was histologically heterogenous, including serous, mucinous, clear cell, and endometrioid subtypes, which may differ in biomarker expression profiles.

Osteopontin is a multifunctional secreted phosphoglycoprotein and has been extensively studied for its role in tumor progression, invasion, angiogenesis, and metastatic potential, particularly in epithelial malignancies [15,17,18]. Experimental studies have shown that osteopontin promotes ovarian cancer cell survival and invasiveness through activation of PI3K/Akt signaling and hypoxia-related pathways, and specific isoforms such as osteopontin-c appear to be selectively expressed in ovarian tumor tissue [15,25]. Despite this strong biological rationale, our findings indicate that circulating osteopontin levels do not reliably distinguish malignant from benign ovarian tumors.

These findings are not in line with previous clinical studies, which suggest that the diagnostic performance of serum osteopontin is broadly comparable to that of CA-125 and expert ultrasonographic evaluation [22]. Clinically, elevated circulating osteopontin levels have been reported in cases with epithelial ovarian cancer compared with healthy controls and benign ovarian disease [19], and meta-analytic data support a positive association between serum osteopontin concentrations and ovarian malignancy in the Asian population [20]. Although some prospective data have reported higher specificity of osteopontin compared with CA-125, particularly in distinguishing malignant from benign ovarian masses, this has generally been accompanied by lower sensitivity, limiting its clinical applicability as a standalone marker [21]. In a meta-analysis of 13 studies with ovarian cancer patients, a sensitivity of 0.66 (95% CI 0.51–0.78) and a specificity of 0.88 (95% CI 0.78–0.93), with an overall AUC of 0.85 has been demonstrated, limiting the utility of serum osteopontin in clinical settings [26]. In some studies, osteopontin has been evaluated as an adjunct to standard diagnostic approaches, with findings suggesting potential usefulness in settings of diagnostic uncertainty, including the differential diagnosis of endometriotic cysts and in less experienced ultrasonographic assessment [22].

One possible explanation for the limited diagnostic performance lies in the distinction between its biological activity within the tumor microenvironment and its detectability in the circulation. Osteopontin primarily acts at the tissue level, where it modulates cell adhesion, invasion, immune interactions, and metastatic behavior [27]. Therefore, this localized activity may not result in sufficiently elevated serum concentrations, particularly in early-stage and non-metastasized disease, thereby limiting its utility as circulation diagnostic biomarker. Studies have demonstrated a correlation between osteopontin expression with advanced tumor stage and poor survival outcomes [28].

A similar pattern was observed for galectin-7, which demonstrated marked interindividual variability and several extreme values, particularly within the benign tumor group. Although benign lesions tended to show higher galectin-7 concentrations, these differences were not statistically significant. Notably, galectin-7 levels were higher in younger and premenopausal women and demonstrated a negative correlation with age, suggesting that circulating galectin-7 may be influenced by demographic or hormonal factors rather than malignancy alone.

These findings are in contrast with tissue-based studies demonstrating that galectin-7 expression is associated with aggressive tumor behavior, high-grade disease, and reduced overall survival in ovarian cancer [23,24]. This discrepancy highlights a key limitation of serum biomarkers: circulating levels may not accurately reflect localized tumor expression, especially when biomarker activity is confined to the tumor microenvironment. Galectin-7 may therefore retain prognostic relevance at the tissue level, while offering limited utility as a circulating diagnostic marker [23].

Overall, our findings add to the growing body of evidence indicating that biologically plausible tumor-associated proteins do not necessarily translate into clinically useful serum biomarkers. The limited performance of osteopontin and galectin-7 observed in this study likely reflects the biological heterogeneity of adnexal masses, overlap between benign and malignant pathophysiological processes, and the multifactorial nature of ovarian tumor biology.

From a clinical perspective, these results reinforce the limitations of relying on single serum biomarkers for the preoperative evaluation of adnexal masses. Despite ongoing investigation of novel markers, CA-125 remains the only FDA-approved serum biomarker for the diagnoses and monitoring ovarian cancer. However, its clinical utility is limited, particularly by poor sensitivity in early-stage disease and lack of specificity [29]. Our findings highlight the importance of multimodal diagnostic strategies that integrate clinical assessment, imaging findings, and biomarker utilization rather than pursuing standalone serum tests.

Future research should aim in evaluating osteopontin and galectin-7 within integraded diagnostic frameworks rather than as isolated circulating markers. An approach could be to combine these biomarkers with advanced imaging techniques, multivariable risk models, or panels of complementary molecular markers. Recent large-scale proteomic analyses have demonstrated marked histotype-specific expression patterns in ovarian cancer [30]. These findings emphasize the importance of tissue-level validation, longitudinal biomarker assessment, and adequately powered population studies stratified by disease stage and histological subtype, to assess performance beyond single-time serum measurements.

The strengths of this study include the prospective study design, the use of a well-defined healthy control group, standardized preoperative biomarker assessment, and comprehensive evaluation of multiple tumor markers within the same population. However, several limitations should be acknowledged. First, this was a single-center design, which may limit external validity. Second, the number of malignant cases was relatively small (n = 19), reducing statistical power and limiting the ability to perform robust subgroup analyses according to the FIGO stage or the histological subtype. Moreover, the malignant cohort was histologically heterogenous and predominantly composed of early-stage disease, which may have influenced circulating biomarker levels and contributed to the limited discriminatory performance observed. Additionally, biomarker measurements were limited to a single preoperative time point, precluding assessment of longitudinal changes. Finally, as most of the malignant cases were epithelial ovarian carcinomas, the findings primarily reflect evidence for the epithelial ovarian cancer and may not be generalizable to other ovarian tumor subtypes.

## 5. Conclusions

Serum osteopontin and galectin-7 do not provide meaningful discriminatory value for differentiating benign from malignant ovarian tumors in a preoperative setting. While both biomarkers remain biologically relevant to ovarian cancer progression, their clinical utility as circulating diagnostic markers appears limited. In contrast, CA-125 remained the only serum biomarker independently associated with malignancy, underscoring its continued clinical relevance. However, the relatively small and predominantly early-stage malignant population limits definitive conclusions regarding the diagnostic performance of osteopontin and galectin-7 across all ovarian cancer types and stages. Larger, stage-diverse studies are needed to assess their potential clinical role. Future research should focus on integrated multimodal approaches to improve risk stratification of adnexal masses.

## Figures and Tables

**Table 1:** Comparison of Age and BMI between cases and controls.

Variable	Group	N	Mean	SD	*t*	*p*-Value
Age (years)	Controls	51	44.27	11.19	−1.391	0.167
	Cases	64	47.83	16.15		
BMI (kg/m^2^)	Controls	51	26.62	6.16	0.582	0.562
	Cases	46	25.93	5.44		

**Table 2:** Comparison of clinical characteristics between cases and controls.

Variable	Category	Controls (N)	Controls (%)	Cases (N)	Cases (%)	χ^2^	*p*-Value
Menopausal status	No	34	66.7	29	47.5	4.129	0.042
	Yes	17	33.3	32	52.5		
Smoking	No	34	66.7	29	54.7	1.554	0.213
	Yes	17	33.3	24	45.3		
Hypertension (HT)	No	45	88.2	46	71.9	4.600	0.032
	Yes	6	11.8	18	28.1		
Diabetes mellitus (DM)	No	50	98.0	60	93.8	1.256	0.262
	Yes	1	2.0	4	6.3		
Parity	No	18	35.3	39	60.9	7.466	0.006
	Yes	33	64.7	25	39.1		

**Table 3:** Demographic and clinical characteristics of the cases.

Variable	No Malignancy (N = 45)	Malignancy (N = 19)	*t*	*p*-Value ^1^
Age (years)	45.6 ± 16.0	53.0 ± 15.8	−1.6948	0.099
BMI (kg/m^2^)	25.5 ± 4.6	27.0 ± 7.4	−0.66079	0.520
Unknown	10	7		—
Values are presented as mean ± standard deviation. ^1^ *p*-values were calculated using Student’s *t* test.				
Variable	No malignancy (N = 45)	Malignancy (N = 19)	Test statistic	*p*-value ^2^
Menopausal status (postmenopausal)	21 (50%)	11 (58%)	χ^2^ = 0.087	0.768
Unknown	3	0		—
Smoking	19 (45%)	5 (29%)	χ^2^ = 0.685	0.407
Unknown	3	2		—
Hypertension	10 (22%)	8 (42%)	χ^2^ = 1.721	0.190
Diabetes mellitus	3 (6.7%)	2 (11%)	-	0.628
Thyroid disease	7 (16%)	2 (11%)	-	0.713
Prior or concurrent malignancy	2 (4.4%)	2 (11%)	-	0.575
Family history of malignancy	3 (6.7%)	2 (11%)	-	0.619

Values are presented as number (%). ^2^ *p*-values were calculated using Pearson’s chi-squared test or Fisher’s exact test, as appropriate.

**Table 4:** FIGO stages of malignant cases.

FIGO Stage	n	%
Stage IA	7	36.7%
Stage IB	3	15.8%
Stage IC	2	10.5%
Stage IIA	1	5.3%
Stage IIB	1	5.3%
Stage IIIA	1	5.3%
Stage IIIB	1	5.3%
Stage IIIC	2	10.5%
Stage IV	1	5.3%

**Table 5:** Comparison of osteopontin and galectin-7 serum levels between controls and cases.

Biomarker	Group	Median	Range	Mann–Whitney U	*p*-Value
Osteopontin (pg/mL)	Controls	14,957.70	4661.4–33,189.3	1529.0	0.562
	Cases	13,046.10	4195.7–39,856.6		
Galectin-7 (pg/mL)	Controls	1330.70	14.0–76,114.4	1895.5	0.138
	Cases	1918.15	250.3–148,528.3		

**Table 6:** Comparison of osteopontin and galectin-7 serum levels between controls, benign, and malignant cases.

Biomarker	Group	Median	Range	Kruskal–Wallis	*p*-Value
Osteopontin (pg/mL)	Controls	14,226.00	4661.4–33,189.3	0.487	0.784
	Benign	13,376.00	4195.7–31,389.8		
	Malignant	12,604.00	4566.2–39,856.6		
Galectin-7 (pg/mL)	Controls	1376.00	14–76, 114.4	3.933	0.140
	Benign	2719.00	250.3–148, 528.3		
	Malignant	1421.00	777.6–4678.4		

**Table 7:** Comparison analysis between osteopontin, galectin-7 and the other biomarkers.

Tumor Marker	Statistic	Osteopontin (pg/mL)	Galectin-7 (pg/mL)
CA-125	Spearman’s rho (ρ)	0.042	0.088
	*p*-value	0.788	0.576
	*N*	43	43
CA 19-9	Spearman’s rho (ρ)	0.070	−0.226
	*p*-value	0.734	0.266
	*N*	26	26
CA 15-3	Spearman’s rho (ρ)	−0.003	0.346
	*p*-value	0.990	0.160
	*N*	18	18
CEA	Spearman’s rho (ρ)	0.225	0.056
	*p*-value	0.216	0.759
	*N*	32	32
AFP	Spearman’s rho (ρ)	−0.096	0.041
	*p*-value	0.686	0.862
	*N*	20	20

## Data Availability

Data are available upon request.

## Appendix A

**Table A1:** Histopathological diagnoses of benign ovarian tumors.

Histopathological Diagnosis	n	%
Serous cystadenoma	16	35.5
Mature cystic teratoma	9	20.0
Mucinous cystadenoma	8	17.7
Endometrioma	5	11.1
Fibroma	1	2.2
Fibroma + Brenner tumor	1	2.2
Ovarian fibroma	1	2.2
Mature cystic teratoma + Benign Brenner tumor	1	2.2
Mucinous cystadenoma + Serous cystadenoma	1	2.2
Serous cystadenoma + Mature cystic teratoma	1	2.2
Atypical hyperplastic serous tumor	1	2.2
Total	45	100

**Table A2:** Histopathological diagnoses of malignant ovarian tumors.

Histopathological Diagnosis	n	%
Serous papillary carcinoma	3	15.8
Brenner tumor	3	15.8
Serous cystadenocarcinoma	2	10.5
Adenocarcinoma	1	5.3
Borderline mucinous carcinoma	1	5.3
Borderline serous carcinoma	1	5.3
Borderline serous carcinoma + Serous cystadenoma	1	5.3
Borderline serous tumor	1	5.3
Clear cell carcinoma	1	5.3
Mature cystic teratoma	1	5.3
Mucinous cystadenocarcinoma	1	5.3
Mucinous cystadenoma with lympatic involvement	1	5.3
Endometrioid ovarian carcinoma	1	5.3
Unknown	1	5.3
Total	19	100

**Table A3:** Surgical management by malignancy status.

Surgical Category/Outcome	No Malignancy (n = 45)	Malignancy (n = 19)
Radical surgery with staging ± lymphadenectomy *	3 (6.7%)	15 (79.0%)
Definitive surgery without staging ^†^	20 (44.4%)	3 (15.8%)
Conservative surgery (ovary-sparing) ^‡^	10 (22.2%)	1 (5.3%)
Ovarian cystectomy only	12 (26.7%)	0 (0%)
Recurrence	0 (0%)	5 (26.3%)
Survival	44 (97.8%)	9 (47.4%)

* Radical surgery with staging includes: total hysterectomy with bilateral salpingo-oophorectomy with surgical staging, with or without lymphadenectomy. ^†^ Definitive surgery without staging includes: total hysterectomy with bilateral salpingo-oophorectomy, subtotal hysterectomy, bilateral salpingo-oophorectomy. ^‡^ Conservative surgery includes: unilateral salpingo-oophorectomy with or without cyst excision or surgical staging.

**Table A4:** Mann–Whitney U tests for osteopontin and galectin-7 according to clinical variables.

Clinical Variable	Statistic	Osteopontin (pg/mL)	Galectin-7 (pg/mL)
Menopausal status (No vs. Yes)	Mann–Whitney U	1843.0	1156.0
	*p*-value	0.079	**0.023**
Smoking (No vs. Yes)	Mann–Whitney U	1079.5	1354.0
	*p*-value	0.158	0.678
Hypertension (No vs. Yes)	Mann–Whitney U	1163.5	1127.5
	*p*-value	0.623	0.807
Diabetes mellitus (No vs. Yes)	Mann–Whitney U	381.0	204.5
	*p*-value	0.146	0.334
Parity (Yes vs. No)	Mann–Whitney U	1647.5	1682.0
	*p*-value	0.975	0.871

**Table A5:** Estimates of the discriminatory performance of osteopontin and galectin-7.

Test Result Variable	Area Under the Curve (AUC)	Standard Error	*p*-Value	95% Confidence Interval (Lower–Upper)
Osteopontin (pg/mL)	0.468	0.054	0.562	0.362–0.575
Galectin-7 (pg/mL)	0.581	0.057	0.138	0.469–0.693
